# The Critical Role of Potassium in Plant Stress Response

**DOI:** 10.3390/ijms14047370

**Published:** 2013-04-02

**Authors:** Min Wang, Qingsong Zheng, Qirong Shen, Shiwei Guo

**Affiliations:** Agricultural Ministry Key Lab of Plant Nutrition and Fertilization in Low-Middle Reaches of the Yangtze River, Jiangsu Key Lab and Engineering Center for Solid Organic Waste Utilization, Nanjing Agricultural University, No. 1 Weigang, Nanjing 210095, China; E-Mails: 2010203034@njau.edu.cn (M.W.); qszheng@njau.edu.cn (Q.Z.); shenqirong@njau.edu.cn (Q.S.)

**Keywords:** biotic stress, abiotic stress, potassium, physiological and molecular mechanisms, plant resistance

## Abstract

Agricultural production continues to be constrained by a number of biotic and abiotic factors that can reduce crop yield quantity and quality. Potassium (K) is an essential nutrient that affects most of the biochemical and physiological processes that influence plant growth and metabolism. It also contributes to the survival of plants exposed to various biotic and abiotic stresses. The following review focuses on the emerging role of K in defending against a number of biotic and abiotic stresses, including diseases, pests, drought, salinity, cold and frost and waterlogging. The availability of K and its effects on plant growth, anatomy, morphology and plant metabolism are discussed. The physiological and molecular mechanisms of K function in plant stress resistance are reviewed. This article also evaluates the potential for improving plant stress resistance by modifying K fertilizer inputs and highlights the future needs for research about the role of K in agriculture.

## 1. Introduction

The world population is expanding rapidly and will pass from its current number of 7.0 billion to 9.4 billion by the year 2050 [1]. To provide enough food for an expanding world population, a massive increase in crop production is required to meet the food demands of future generations, while preserving the ecological and energy-related resources of our planet. However, agricultural production continues to be constrained by a variety of biotic (e.g., pathogens, insects and weeds) and abiotic (e.g., drought, salinity, cold, frost and waterlogging) factors that can significantly reduce the quantity and quality of crop production. Evidence indicates that biotic stress can cause a 28.2% yield loss of wheat, 37.4% loss of rice, 31.2% loss of maize, 40.3% loss of potatoes, 26.3% loss of soybeans and 28.8% loss of cotton [2]. Meanwhile, yield losses from abiotic stress were estimated at 65.8% for maize, 82.1% for wheat, 69.3% for soybeans and 54.1% for potatoes [3].

During their evolution, plants have developed a wide range of mechanisms to resist a variety of stressed conditions. Increasing evidence suggests that mineral nutrients play a critical role in plant stress resistance [4–8]. Out of all the mineral nutrients, potassium (K) plays a particularly critical role in plant growth and metabolism, and it contributes greatly to the survival of plants that are under various biotic and abiotic stresses. The importance of K fertilizer for the formation of crop production and its quality is known. As a consequence, potash consumption has increased dramatically in most regions of the world [9]. A strong positive relationship between K fertilizer input and grain yield has been shown [10].

K is an essential nutrient and is also the most abundant cation in plants. The concentration of K^+^ in the cytoplasm has consistently been found to be between 100 and 200 mM [11], and apoplastic K^+^ concentration may vary between 10 and 200 or even reach up to 500 mM [12]. K plays essential roles in enzyme activation, protein synthesis, photosynthesis, osmoregulation, stomatal movement, energy transfer, phloem transport, cation-anion balance and stress resistance [4].

This review is focused on the effects of K on plant resistance to various biotic (pathogen and insects) and abiotic (drought, salinity, cold and frost and waterlogging) stresses. K availability for plant growth, anatomy and morphology, as well as plant metabolism are discussed. This review also discusses the roles of K in stress-resistant mechanisms and evaluates the potential for improving plant resistance by modifying K fertilizer input and selecting appropriate plant species or varieties.

## 2. The Role of Potassium in Biotic Stress Resistance

Increased evidence has shown that crop production is significantly restricted by biotic stresses. Oerke and Dehne [13] estimated that weeds produce the highest potential loss (32%), followed by animal pests (18%), fungi and bacteria (15%) and viruses (3%) from 1996 to 1998. These numbers reflect the total attainable production for eight major crops (wheat, rice, maize, barley, potatoes, soybeans, sugar beets and cotton). In many cases, K-deficient plants tend to be more susceptible to infection than those with an adequate supply of K. For example, the rate of rice borer infestation was greatest when there was no supply of K, but decreased rapidly as the K concentration increased [14] (Table 1). Similar results were found with a *Discula destructiva* Redlin infection in *Cornus florida* L. [15]. Williams and Smith [16] also reported that increased K fertilizer significantly reduced the disease incidence of stem rot and aggregate sheath spot (AgSS), and negative correlations were found between the percentage of K in leaf blades and disease severity. K fertilizer is widely reported to decrease insect infestation and disease incidence in many host plants. Perrenoud [17] reviewed 2449 references and found that the use of K significantly decreased the incidence of fungal diseases by 70%, bacteria by 69%, insects and mites by 63%, viruses by 41% and nematodes by 33%. Meanwhile, K increased the yield of plants infested with fungal diseases by 42%, bacteria by 57%, insects and mites by 36%, viruses by 78% and nematodes by 19%.

K fertilizer application decreased the incidence of diseases in most cases, but sometimes had no effect or even the opposite effect. Prabhu *et al.*[18] noted that the effect of K on disease incidence can be classified as “increased”, “decreased” and having “no effect” or “variable effect” (Table 2). The variable effects of K on disease incidence could be affected by the amount and source of K, plant and pathogen species and trial type. Nam *et al.*[19] found that strawberries that were grown with excess K were very susceptible to infection by the anthracnose pathogen, *Colletotrichum gloeosporioides*, but its resistance was greatly enhanced when no K was supplied. This result was observed because the low plant K status induces the synthesis of molecules, including reactive oxygen species (ROS) and phytohormones, such as auxin, ethylene and jasmonic acid (JA), as a result of its enhanced plant stress tolerance [5,20].

The mechanistic influences of K on plant disease resistance have been reported by several researchers. Higher K^+^ concentrations decreased the internal competition of pathogens for nutrient resources [15]. This nutritional status enables plants to allocate more resources to developing stronger cell walls for preventing pathogen infection and insect attack and to obtain more nutrients to be used for plant defense and damage repair [21]. DeDatta and Mikkelson [22] reported that the culm and stalk strength of rice were increased in the presence of adequate K concentrations as a result of increasing plant resistance. During airborne pathogen infections (especially from bacteria and viruses), the stomata were able to function properly when there was sufficient K, thus preventing pathogen invasion by rapid stomata closing [23,24].

**Figure 1:**
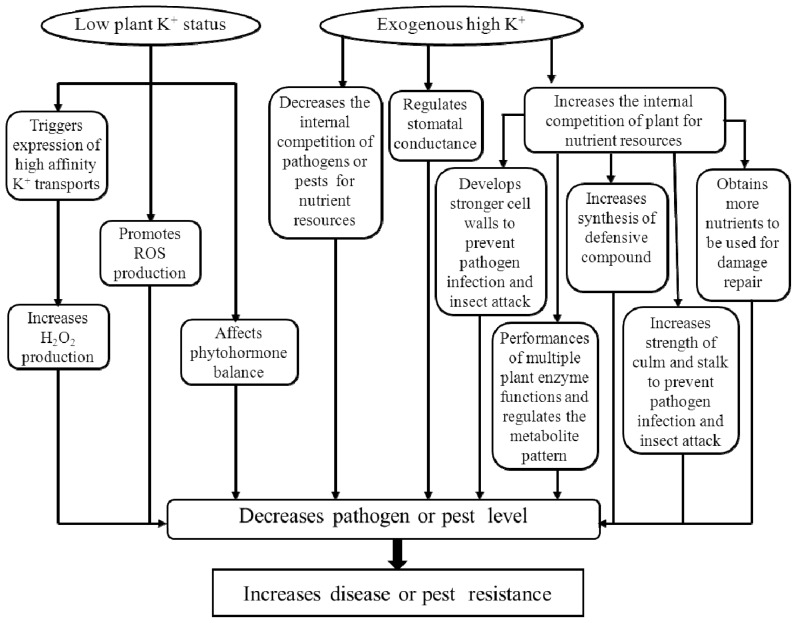
Role of K under biotic stress.

K is also essential to the performance of multiple plant enzyme functions, and it regulates the metabolite pattern of higher plants, ultimately changing metabolite concentrations [4,21]. In a K-sufficient plant, the synthesis of high-molecular-weight compounds (such as proteins, starches and cellulose) was markedly increased, thereby depressing the concentrations of low-molecular-weight compounds, such as soluble sugars organic acids, amino acids and amides, in the plant tissues. These low-molecular-weight compounds are important for the development of infections and insect infestations, so lower concentrations, thereby, leave plants less vulnerable to disease and pest attacks in K-sufficient plants [4]. Adequate K increases phenol concentrations, which play a critical role in plant resistance [25]. Furthermore, Sarwar [14] concluded that less pest damage in higher K plants can be attributed to a lack of pest preference under sufficient nutrient concentrations, as well as the synthesis of defensive compounds leading to higher pest mortality. Figure 1 summarizes the main roles of K in plant resistance to biotic stress.

## 3. The Role of Potassium in Abiotic Stress Resistance

### 3.1. Potassium and Drought Stress

The major limitation for plant growth and crop production in arid and semi-arid regions is soil water availability. Plants that are continuously exposed to drought stress can form ROS, which leads to leaf damage [7,13,26] and, ultimately, decreases crop yield. During drought stress, root growth and the rates of K^+^ diffusion in the soil towards the roots were both restricted, thus limiting K acquisition. The resulting lower K concentrations can further depress the plant resistance to drought stress, as well as K absorption. Maintaining adequate plant K is, therefore, critical for plant drought resistance. A close relationship between K nutritional status and plant drought resistance has been demonstrated. The roles of K in physiological and molecular mechanisms of plant drought resistance have been explored.

#### 3.1.1. Cell Elongation and Cell Membrane Stability

It is worthwhile to try to improve crop tolerance to stress in low-moisture soils by inducing deeper rooting, larger absorption surfaces and greater water retention in plant tissues. Deeper rooting could be achieved by deep placement of K fertilizer that is associated with other mineral nutrients, such as P and N, which both have root signaling functions [27]. Adequate amounts of K can enhance the total dry mass accumulation of crop plants under drought stress in comparison to lower K concentrations [28]. This finding might be attributable to stomatal regulation by K^+^ and corresponding higher rates of photosynthesis [4]. Furthermore, K is also essential for the translocation of photoassimilates in root growth [6]. Root growth promotion by increased appropriate K supply under K-deficient soil was found to increase the root surface that was exposed to soil as a result of increased root water uptake [6]. Lindhauer [29] reported that fine K nutrition not only increased plant total dry mass and leaf area, but also improved the water retention in plant tissues under drought stress.

Increased evidence shows that the maintenance of membrane integrity and stability under drought stress is also essential for plant drought tolerance [30]. Cell membrane stability was significantly declined under drought stress [31]. In a study by Premachandra *et al.*[32], maize plants with higher K applications showed greater adaptation to water stress. This improvement was mainly attributed to the role of K in improving cell membrane stability and osmotic adjustment ability. An adequate K supply is essential to enhancing drought resistance by increasing root elongation and maintaining cell membrane stability.

#### 3.1.2. Aquaporins and Water Uptake

Aquaporins are channel proteins that are present in the plasma and intracellular membranes of plant cells. They play a crucial role in plant water relations by regulating the osmotic potential and hydraulic conductivity of membranes and make changes in plant water permeability [33,34]. Under drought stress conditions, aquaporin gene expression can be regulated [35,36] to help plants maintain their water balance [36–38].

During water stress, roots regulated their water and ion uptake capacities by modifying *PIPs* (plasma membrane intrinsic proteins) and K^+^ channel at the transcription level to cope with the water deficiency [37,39–42]. Liu *et al.*[41] observed that transcription expression levels of the *PIPs* and K^+^ channel-encoding genes was induced by K^+^ starvation and could be downregulated by a polyethylene glycol (PEG)-mediated water deficit in rice, which may result in a reduction in the membrane water permeability and then promote cellular water conservation during drought stress. However, it should be mentioned that the expression level of some other water channels, such as OsPIP1;1, OsPIP1;2, AtPIP1;4 and AtPIP2;5, could be induced by a relative long-term water-deficit treatment, which should result in greater osmotic water permeability and facilitated water flux [40,41]. Recently, Kanai *et al.*[43] also observed close coupling between aquaporin activities and K-channel transporters. The initial response of K deficiency was perceived in the form of a change in K-channel activity, which altered root hydraulic conductance, and transduction of the follow up signal resulted in a shift of aquaporin activity. They found that aquaporin activities might have been suppressed by K deficiency and result in a reduction of root hydraulic conductance and water supply to the growing stem for diameter expansion and the leaf for transpiration.

In *Arabidopsis* roots, the transcripts encoding water channels, PIP1;2 (PIP1b), PIP2;2 (PIP2b) and TIP1;2 (TIP), and the K^+^ transporter, HAK5, were reduced under treatment of the K^+^-channel blocker (CsCl) [44]. Furthermore, water transport in onion roots was sensitive to inhibitors of the water channel and K^+^-channel, and the reduction in hydraulic conductivity (*Lp*) by treatment with a K^+^-channel inhibitor suggests that K^+^ fluxes are linked to water channel activity in the plasma membrane [45]. Water channels and K^+^ channel/transporters are functionally co-regulated as a part of plant osmoregulation to maintain appropriate cytosolic osmolarity and acclimate the plant to drought or other stresses [41]. Guo *et al.*[46] also showed a positive correlation between K absorption and water uptake in *Phaseolus vulgaris* plants. The loading of K^+^ into the xylem most likely mediated the xylem hydraulic conductance that aided plants in maintaining cell turgor, stomatal aperture and gas exchange rates as part of their drought adaptations [47,48].

#### 3.1.3. Osmotic Adjustment

The maintenance of a favorable water status is critical for plant survival under drought stress. Osmotic adjustment is a major trait that is associated with maintaining high cellular turgor potential and water retention in response to drought stress. Many studies have shown that osmotic adjustment of leaves is positively correlated with drought tolerance in various plant species [49]. As one of the most prominent inorganic osmotica in plants, K^+^ plays a key role in formation of the osmotic adjustment ability, even under drought conditions [4]. Cell turgor recovery in osmotically-generated stress was regulated by increasing K^+^, Cl^−^ and Na^+^ uptake by root cells, which was partly mediated by voltage-gated K^+^ transporters at the cellular plasma membrane [50]. Furthermore, sufficient K induces solute accumulation, thus lowering osmotic potential and helping to maintain plant cell turgor under osmotic stress. In summary, an adequate K status may facilitate osmotic adjustment, which maintains higher turgor pressure, relative water content and lower osmotic potential, thus improving the ability of plants to tolerate drought stress [8,51].

#### 3.1.4. Stomatal Regulation

One of the major functions of the stomata is to control plant water loss via transpiration. During drought stress, quick stomatal closure and internal moisture preservation are essential for plant adaptation to drought conditions. K plays a crucial role in turgor regulation within the guard cells during stomatal movement [4]. As stomatal closure is preceded by a rapid release of K^+^ from the guard cells into the leaf apoplast, it is reasonable to think that stomata would be difficult to remain open under K-deficient conditions. Some studies also stated that K deficiency may induce stomatal closure and inhibit photosynthetic rates in several crop plants [52,53]. Conversely, many studies suggest that K had no effect on stomatal conductance and photosynthetic rates under well-watered conditions, but K starvation could favor stomatal opening and promote transpiration, compared with K sufficiency in several plants under drought stress [54–56]. Furthermore, photosynthetic rate was decreased under drought stress in K-deficient plants [51,54,57]. This discrepancy may be related to the plant species, experimental system and environmental factors within the experimental field or interspecific differences.

The effects of drought stress on stomata closure in olive trees and sunflower plants were found to be dependent on the K^+^ nutrient status [55,56]. When plants were supplied with different K^+^ concentrations and then subjected to drought stress, their stomatal conductance was more markedly reduced in normal K plants than in low K plants (Table 3). Benlloch-Gonzalez *et al.*[56] explained that the low plant K status could inhibit water-stress-induced stomatal closure via ethylene synthesis, and stomatal conductance could be significantly reduced in K^+^-starved plants after the adding of an ethylene synthesis inhibitor (cobalt). K^+^ starvation increases the transcription of genes involved in ethylene production and signaling and stimulates ethylene production [56,58]. Then, the increased ethylene could inhibit the action of abscisic acid (ABA) on stomata and delay stomata closure [59,60]. During drought stress, the stomata cannot function properly in K^+^-deficient plants, resulting in greater water loss. Drought stress did not decrease water use efficiency (WUE), whereas it did increase WUE by rapid stomata closing during water deficit [51]. Adequate levels of K nutrition enhanced plant drought resistance, water relations, WUE and plant growth under drought conditions [51].

#### 3.1.5. Detoxification of Reactive Oxygen Species

Stomatal closing in response to drought stress leads to a reduction in photosynthetic efficiency as a consequence of chloroplast dehydration [7]. Photosynthesis inhibition can further disturb the balance between ROS production and antioxidant defense [61–63], resulting in ROS accumulation. The ROS have a dual action in biotic and abiotic stresses that depends on their cellular concentration [64]. Low levels of ROS could be involved in the stress-signaling pathway by triggering stress defense/acclimation responses [64,65]. However, ROS became extremely injurious to cellular membranes and other cellular components when its concentrations reached the point of phytotoxicity, resulting in oxidative stress and, eventually, cell death [64,66].

Drought stress-induced ROS production can additionally be enhanced in K-deficient plants [7]. Under drought stress, photosynthetic CO_2_ fixation in K-deficient plants is substantially limited by impairment in stomata regulation, conversion of light energy into chemical energy and phloem export of photosynthates from source leaves into sink organs [51]. As the impairment in photosynthetic CO_2_ fixation occurs, molecular O_2_ is activated, leading to extensive generation of ROS [67] and, thereby, oxidative degradation of chlorophyll and membranes. The maintenance of adequate K nutrition is critical for mitigating or preventing damage by drought stress and controlling the water balance [68]. Egilla [51] suggested that increasing extrachloroplastic K^+^ concentrations in plant cells with an excess K^+^ supply could prevent photosynthesis inhibition under drought stress. An adaptive K requirement for drought-stressed plants could be related to the role of K in enhancing photosynthetic CO_2_ fixation and transport of photosynthates into sink organs and inhibiting the transfer of photosynthetic electrons to O_2_, thus reducing ROS production [7].

Beside the photosynthetic electron transport, nicotinamide adenine dinucleotide phosphate (NADPH)-dependent oxidase activation represents another major source for production of ROS in plant cells by a number of biotic and abiotic stress factors [65]. NADPH-oxidizing enzymes catalyze one-electron reduction of O_2_ to O_2_^•−^ by using NADPH as an electron donor [7]. Cakmak [7] reported that activity of NADPH oxidase was increased in cytosolic fractions of bean roots with increasing severity of K deficiency, resulting in an increase in NADPH-dependent O_2_^•−^ generation. The reason for the increase of NADPH oxidase by K deficiency is probably that K deficiency induced ABA accumulation [69]. Furthermore, ABA has also been shown to be effective in increasing H_2_O_2_ and O_2_^•−^ accumulations in roots or leaves [70,71], but this point needs to be clarified in future studies. An improvement in the plant K supply can inhibit ROS production under drought stress by reducing NADPH oxidase activity and maintaining photosynthetic electron transport [7]. In addition to K, various micronutrients, including Zn, B, Cu and Mn, have also been shown to be involved in detoxifying oxygen radicals [72]. The K supply is thus associated with other mineral nutrients and is essential for the detoxification of active oxygen under drought stress.

**Figure 2:**
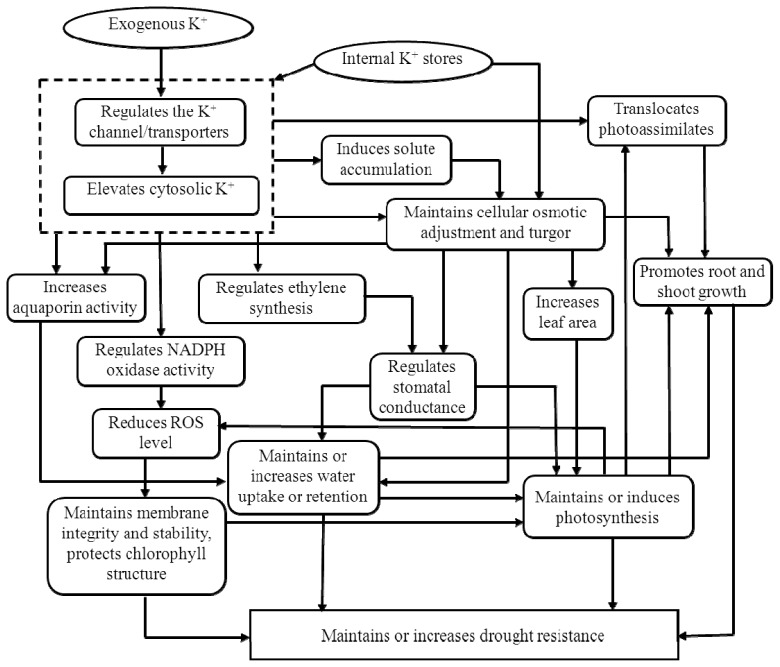
Role of K under drought stress.

In brief, a sufficient K status increased cell membrane stability, root growth, leaf area and total dry mass for plants living under drought conditions and also improved water uptake and water conservation. Maintaining an adequate K nutritional status is critical for plant osmotic adjustment and for mitigating ROS damage as induced by drought stress. In Figure 2, we summarize the role of K in plants that are living under drought stress.

### 3.2. Potassium and Salt Stress

Salinity is a major abiotic stress that affects approximately 7% of the world’s total land area. More than 800 million hectares of land around the world are affected by salinity [73], which results in billions of dollars in crop production losses. The accumulation of high salt concentrations in the soil makes it harder for plant roots to take up water and, thereby, disturbs a plant’s water balance, while high concentrations of salts in plant tissue may be toxic. Salinity inhibits seed germination and plant growth, affects the leaf anatomy and physiology of plants and, thereby, influences their photosynthesis, water relations, protein synthesis, energy production and lipid metabolism [74]. Plant growth responds to salinity in the following two phases [75]: a rapid osmotic phase that decreases water availability to plants and inhibits the growth of young leaves, followed by a slow ionic phase that results in salt toxicity and accelerates the senescence of mature leaves. Plants have developed diverse strategies to resist salt stress, such as restricting Na^+^ uptake, activating Na^+^ exclusion or cellular compartmentalization of excessive Na^+^ into the vacuole [76,77].

Salt-stressed root growth is restricted by osmotic effects and toxic effects of ions, which results in lower nutrient uptake and inhibits the translocation of mineral nutrients, especially K^+^. As a result of the similarities in physicochemical properties between Na^+^ and K^+^, Na^+^ could compete with K^+^ for major binding sites in key metabolic processes, including both low-affinity (e.g., non-selective cation channels (NSCC)) and high-affinity (e.g., KUP and high-affinity K^+^ transporter (HKT)) transporters and could also disturb plant metabolism [4,78]. K^+^ deficiency can usually be observed under salinity stress. First, high levels of Na^+^ inhibit K^+^ activity in the soil solution, resulting in a reduction of K^+^ availability. Second, Na^+^ not only interferes with K^+^ translocation from root to shoot (especially in low K^+^ status) [79], but also competes with K^+^ for uptake sites at the plasma membrane, resulting in lower K^+^ uptake. Third, salinity stress leads to plasma membrane dis-integrity and favors K^+^ leaking, resulting in a rapid decline in cytosolic K^+^[80]. Also, salinity induces significant membrane depolarization and favors K^+^ leaking through depolarization-activated outward-rectifying (KOR) K^+^ channels [78]. Therefore, keeping cellular K^+^ content above a certain threshold and maintaining a high cytosolic K^+^/Na^+^ ratio (either by retaining K^+^ or preventing Na^+^ from accumulating in the leaves) is critical for plant growth and salt tolerance. An increasing K supply corresponded with higher K^+^ accumulation in plant tissue, which reduced the Na^+^ concentration and resulted in a higher K^+^/Na^+^ ratio. Members of the HKT transporter (high-affinity K^+^ transporter) family that mediate Na^+^-specific transport or Na^+^-K^+^ co-transport play a key role in plant Na^+^ tolerance mechanisms [81,82]. HKT represents a primary mechanism in the regulation of Na^+^ and K^+^ homeostasis, as well as Na^+^ exclusion [83,84].

Plant growth and salt tolerance were sharply reduced when exposed to a combination of salt stress and K-deficiency stress. K^+^ deficiency significantly increased the negative effects that were induced by salt in the photosynthesis of barley and was accompanied by an increase in salt sensitivity [85]. Similar results were found by Qu *et al.*[86,87], in which K^+^ deficiency significantly inhibited nitrogen and photosynthetic carbon assimilation and also impaired the light reaction pathways of PS I and PS II in maize under salt stress. In a study by Chen *et al.*[88], K^+^ flux from barley root in response to NaCl treatment was highly positively correlated with net CO_2_ assimilation, plant growth, survival rate, relative grain yield and tolerance to salt stress (Table 4).

Increased evidence has shown that K can involve osmotic adjustment of salt-stressed plants. During salt stress conditions, increased Na^+^ concentrations were accumulated in the vacuole and a substantial osmotic potential gradient was established between the vacuole and the cytosol by depressing the cytosol’s water activity. This change requires a coordinated increase in compatible solutes in the cytosol to balance out the osmotic pressure. Munns and Tester [75] reviewed that plants have a Na^+^ exclusion mechanism that maintains a low level of Na^+^ in the leaves during salt stress; thus, the major osmoticum in leaves was K^+^. K^+^ plays an important role in maintaining cell turgor and osmotic adjustment. The vacuole and the cytosol are the two major pools of K in plant cells. Cytosolic K^+^ concentrations are maintained at a constant level and are essential for plant metabolism, while vacuolar K^+^ concentrations may vary dramatically. Under K^+^-deficient conditions, a constant cytosolic K^+^ concentration was attributed to the consumption of vacuolar potassium [89].

**Figure 3:**
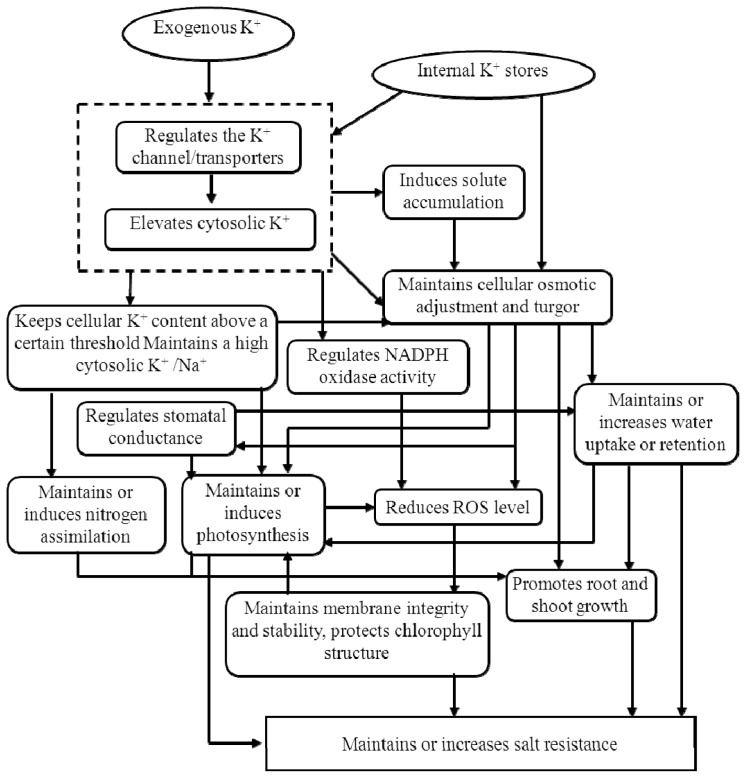
Role of K under salt stress.

Low K^+^ status might induce the formation of ROS and related cell damage under saline conditions, which was attributed to the effects of K^+^ deficiency and/or Na^+^ toxicity on stomatal closing and the inhibition of photosynthetic activity and ultimately inhibits plant growth and reduces crop production [90]. Previous articles have shown that salinity-induced ROS formation can lead to programmed cell death (PCD), and a high cytosolic K^+^/Na^+^ ratio is essential for triggering salinity-induced PCD [91]. A decrease in the cytosolic K^+^ pool would activate caspase-like proteases and lead to PCD under saline conditions. The ability of plants to satisfy their metabolic requirements for K^+^ in the presence of salinity by using higher K^+^ fluxes and lower Na^+^ fluxes that result in a higher K^+^/Na^+^ selectivity ratio is essential for salt tolerance. The addition of K^+^ to a saline culture solution has been found to increase K^+^ concentrations in plant tissue that corresponds with a decrease in Na^+^ content, with a further increase in plant growth and salt tolerance. Increased evidence shows that it is not the absolute quantity of Na^+^*per se* that influences salt resistance, but rather the cytosolic K^+^/Na^+^ ratio that determines plant salt tolerance [11,78]. Figure 3 summarizes the role of K in plants living under salt stress.

### 3.3. Potassium and Low-Temperature Stress

Cold stress inhibits plant growth and development, which results in limited crop productivity. It affects plants by directly inhibiting metabolic reactions and indirectly influencing cold-induced osmotic, oxidative and other stresses. The effect of increasing K^+^ applications on yield and cold tolerance studied by Devi *et al.*[92] in *Panax ginseng* showed that a high K^+^ concentration activated the plant’s antioxidant system and increased levels of ginsenoside-related secondary metabolite transcripts, which are associated with cold tolerance. Cold stress may destroy photosynthetic processes and reduce the effectiveness of antioxidant enzymes, resulting in ROS accumulation [66,93,94]. K improved plant survival under cold stress by increasing antioxidant levels and reducing ROS production [7,92].

Greater frost damage in K-deficient plants is related to water deficiency from the chilling-induced inhibition of water uptake and freezing-induced cellular dehydration [95]. A significant negative correlation was found between frost damage and leaf K concentration, and an adequate K supply can effectively increase frost resistance [6,8]. Bogdevitch [96] found that oats that were supplied with sufficient K could survive late frost without obvious damage, whereas much of the crop that was grown on K-deficient soil did not survive. This finding could be attributed to a regulation of osmotic and water potential and a reduction of electrolyte leakage caused by cold stress [8,97]. High concentrations of K^+^ protected against freezing by lowering the freezing point of the plant’s cell solution. Furthermore, an adapted cytosol K^+^ concentration is also essential for enzyme activities that are involved in regulating frost resistance [8].

Because the plasma membrane is the primary site for perceiving changes in temperature, membrane fluidity can be decreased by cold stress as a result of changes in fatty acid unsaturation and the lipid-protein composition of the cell membrane [98]. The ratio of unsaturated/saturated fatty acids in the cell membrane was essential for plant cold tolerance, and the higher the ratio in the cell membrane, the more tolerant the tissue is to cold stress [99]. A decrease in membrane fluidity could further affect the transport of ions, water and metabolites. The effects of nitrogen and potassium on spikelet sterility induced by low temperature at the reproductive stage of rice were studied by Haque [100]. The spikelet sterility induced by low temperature was decreased with the increase of K^+^ supply and the increase of the K/N ratio in the rice leaves. Increasing plant frost resistance by the addition of K is associated with the increase in phospholipids, membrane permeability and improvement in the biophysical and biochemical properties of cell [101].

**Figure 4:**
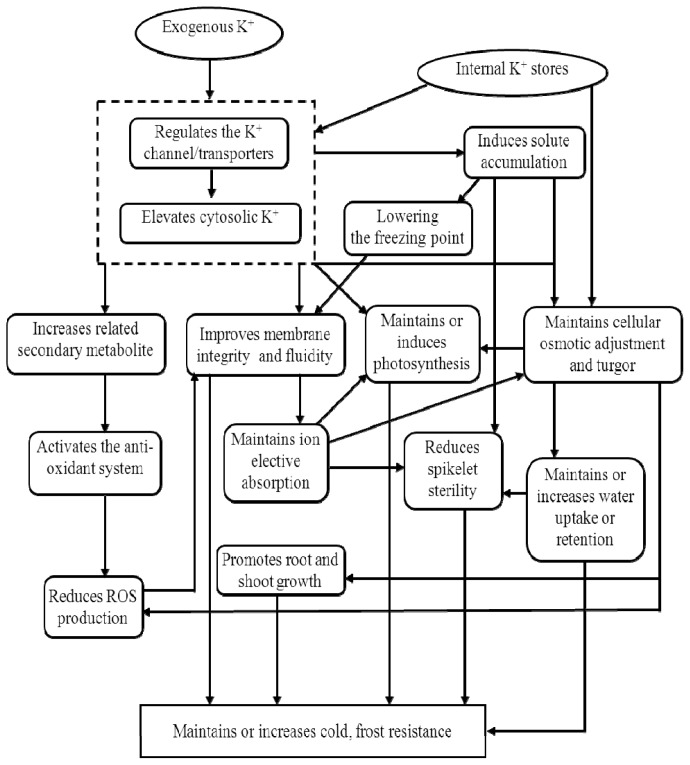
Role of K under cold and frost stress.

In brief, higher K tissue concentrations reduced chilling damage and increased cold resistance, ultimately increasing yield production [8,21]. Frost damage was inversely related to K concentration and was significantly reduced by K fertilization. Figure 4 summarizes the role of K in the plant under low temperature stress.

### 3.4. Potassium and Waterlogging Stress

Waterlogging affects approximately 10% of the global land area [102] and is a serious impediment for sustainable agriculture development. Yield losses due to waterlogging may vary between 15% and 80%, depending on the crop species and growth stage, soil type and duration of the stress [103], resulting in severe economic penalties in some area. The important biological consequence of waterlogging is that the respiration of roots and micro-organisms depletes the residual oxygen and the environment becomes hypoxic (*i.e.*, oxygen levels limit mitochondrial respiration) and, later, anoxic (*i.e.*, respiration is completely inhibited) [104,105]. The low energy status under oxygen deficient conditions results in a substantial depolarization of plasma membrane potential [106], subsequent impairment of ion transport processes through voltage-gated uptake channels and a decrease of the uptake of most essential cations (e.g., K^+^, NH_4_^+^ or Mg^2+^) [107,108]. Pang *et al.*[109] reported that hypoxia-induced K^+^ flux responses are mediated by both inwardly rectifying potassium (KIR) and NSCC channels in the elongation zone, while KOR channels in the mature zone are likely to play a critical role. Avoiding K^+^ loss during hypoxia or anoxia stress is the key mechanism responsible for waterlogging resistance in plants [109–112].

Furthermore, as flooding time increases, potentially toxic compounds, such as sulfides, soluble iron and manganese, ethanol, CO_2_, ethylene, lactic acid, acetaldehyde and acetic and formic acid, were accumulated as the result of the reduced soil redox potential [106,113]. Those compounds acted on cellular membranes, leading to phospholipid oxidation and a subsequent change in membrane integrity and membrane transport [109,114]. Rapid changes in net K^+^ were measured in response to the application of secondary metabolites (various monocarboxylic acids and phenolic acids) produced by waterlogged soils [115]. Shabala [106] assumed that organic acid uptake across the plasma membrane results in a net H^+^ influx and causes a substantial membrane depolarization. Such a depolarization will significantly affect intracellular K^+^ homeostasis by reducing K^+^ uptake via KIR, as well as enhancing K^+^ efflux via KOR.

Waterlogging is known to block the oxygen supply to the roots, thus inhibiting root respiration, resulting in a severe decline in energy status of root cells, affecting important metabolic processes of plants. Under waterlogged conditions, the stomata conductance, photosynthesis rate and root hydraulic conductivity of plant were hampered [116]. The oxidative damage induced by the generation of reactive oxygen species affects the integrity of membranes and induces damage to the efficiency of photosystem II, thereby, causing a considerable decrease in net photosynthetic rates [117]. Exogenous application of K could effectively ameliorate the adverse effects of waterlogging on plants. K supplement under waterlogging not only increased plant growth, photosynthetic pigments and photosynthetic capacity, but also improved plant nutrient uptake as a result of higher K^+^, Ca^2+^, N, Mn^2+^ and Fe^2+^ accumulation [118]. Ashraf *et al.*[118] also reported that exogenous application of K in soil and as foliar spray alleviated the adverse effects of waterlogging on cotton plants.

## 4. Implications

**Figure 5:**
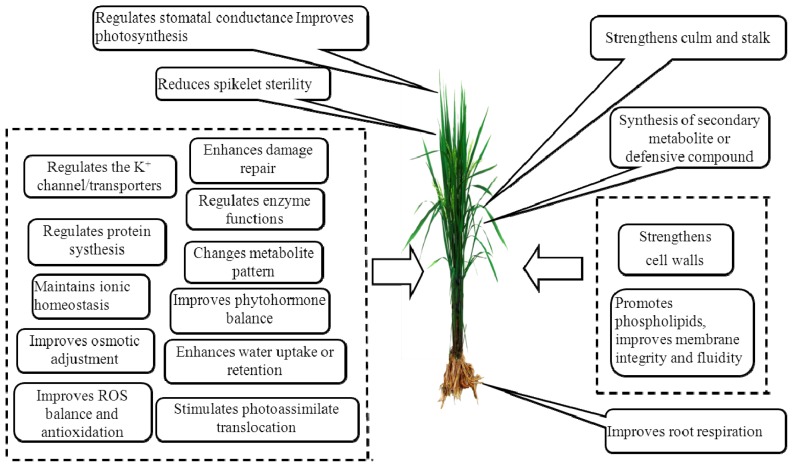
Roles of K in resisting all stresses.

The population of the world will exceed 9 billion by the year 2050. It is, therefore, of vital importance to improve crop yield to match the requirement for food. However, as the environment was becoming worse, the quantity and quality of crop production were significantly decreased by a variety of biotic and abiotic stresses. The practice of intensive fertilization to support massive food production for an increasing global population is a must. However, consumption of excess N fertilization and K deficiency cause a reduction in crop yields and quality in many regions. Therefore, to enable closing yield gaps and allow for a much higher productivity in many regions, a significant increase in K fertilization application is required. K is an essential plant nutrient that impacts a number of physiological and biochemical processes that are involved in plant resistance to biotic and abiotic stresses, as summarized in Figure 5.

Maintaining an optimum K nutritional status is essential for plant resistance to biotic and abiotic stresses. Balanced fertilization and efficient K usage in combination with other nutrients not only contribute to sustainable crop’s growth, yield and quality, but also influence plant health and reduce the environmental risks. However, our understanding about the role of K in whole-plant stress response mechanisms is limited. In this paper, suggested future needs and prospects for research about the role of K in agriculture include:

1. Investigating more details about the molecular mechanisms of K in plant stress resistance.
2. Examining the role of K on plant resistance to biotic and abiotic stresses in differentiated cells, tissues and organs and connecting the data relevantly.
3. Identifying the common or specific response of K to distinct stress and the role of K on long-term plant responses under multiple stress conditions in nature.
4. Understanding the relationship between K and other nutrients in relation to plant adaptation to stresses in different agroecological systems.
5. Developing models for better K recommendations based on soil, plant and environmental factors.
6. Investigating more researcher on the importance of K on crop production, nutritional quality and human and animal health.

## Figures and Tables

**Table 1:** Impacts of soil potassium build-up on rice stem borers and grain yield within a rice field [14].

Serial number	Potassium treatments (kg ha^−1^)	Stem borer infestation (%)		Yield/plot (g/3 m^2^)	Yield (kg ha^−1^)

		Dead heart	White heads		
1	40 kg	3.05 b	5.37 b	1913.00 b	6376.66
2	50 kg	2.64 bc	3.58 c	287.00 a	7623.33
3	60 kg	2.40 c	3.37 c	2317.00 a	7723.33
4	Control	4.33 a	7.12 a	1690.00 c	5633.33
LSD value		0.619	0.561	219.4	

The means in each column are followed by at least one letter in common and are not significantly different at the 5% level of probability (*p* ≥ 0.05). LSD, least significant difference.

**Table 2:** Number of published papers reporting the effects of potassium on disease [18].

	Decrease in disease	Increase in disease	No effect	Total
Fungi	89	33	8	130
Bacteria	18	5	0	23
Viruses	9	5	3	17
Nematode	3	6	1	10

**Table 3:** Effect of K^+^ concentration in irrigation water (normal *versus* low K) and water availability in the growth medium (irrigation *versus* drought) on K^+^ accumulation and stomatal conductance in leaves [56].

Treatment	K^+^ content in leaves (μmol/gFW)	Stomatal conductance (mmol/m^2^/s)
Normal K: Irrigated	133.6 ± 7.3	456 ± 5.6
Normal K: Drought	119.4 ± 3.8	281 ± 27.9
Low K: Irrigated	36.3 ± 1.4	462 ± 4.0
Low K: Drought	25.7 ± 0.8	351 ± 15.2

**Table 4:** Linear correlation (*r* values) between various physiological characteristics from 62 barley genotypes in glasshouse and laboratory experiments [88].

Parameter	Grain yield	K^+^ flux	Shoot biomass	Survival rate	Plant height	[CO_2_]ass	TSW	Tillering
K^+^ flux	0.67 **	-	-	-	-	-	-	-
Shoot biomass	0.96 **	0.69 **	-	-	-	-	-	-
Survival rate	0.65 **	0.70 **	0.74 **	-	-	-	-	-
Plant height	0.70 **	0.69 **	0.61 **	0.51 **	-	-	-	-
[CO_2_]ass	0.68 **	0.69 **	0.65 **	0.48 **	0.50 **	-	-	-
TSW	0.72 **	0.70 **	0.70 **	0.63 **	0.74 **	0.48 **	-	-
Tillering	0.48 **	0.26 *	0.51 **	0.16	0.23	0.25 *	0.33 *	-
Germination	0.29 *	0.21	0.31 *	0.33 **	0.16	0.02	0.38 **	0.20

** *p* < 0.01;

* *p* < 0.05;

TSW: thousand-seed weight; [CO_2_]ass: CO_2_ assimilation.
